# Integrated analysis revealing a novel stemness-metabolism-related gene signature for predicting prognosis and immunotherapy response in hepatocellular carcinoma

**DOI:** 10.3389/fimmu.2023.1100100

**Published:** 2023-08-09

**Authors:** Yuxin Wang, Xueshuai Wan, Shunda Du

**Affiliations:** Department of Liver Surgery, Peking Union Medical College Hospital, Chinese Academy of Medical Sciences and Peking Union Medical College (CAMS & PUMC), Beijing, China

**Keywords:** mRNAsi, cancer stem cell, metabolism reprogramming, machine learning, immunotherapy, prognosis, hepatocellular carcinoma

## Abstract

Hepatocellular carcinoma (HCC) is a malignant lethal tumor and both cancer stem cells (CSCs) and metabolism reprogramming have been proven to play indispensable roles in HCC. This study aimed to reveal the connection between metabolism reprogramming and the stemness characteristics of HCC, established a new gene signature related to stemness and metabolism and utilized it to assess HCC prognosis and immunotherapy response. The clinical information and gene expression profiles (GEPs) of 478 HCC patients came from the Gene Expression Omnibus (GEO) and the Cancer Genome Atlas (TCGA). The one-class logistic regression (OCLR) algorithm was employed to calculate the messenger ribonucleic acid expression-based stemness index (mRNAsi), a new stemness index quantifying stemness features. Differentially expressed analyses were done between high- and low-mRNAsi groups and 74 differentially expressed metabolism-related genes (DEMRGs) were identified with the help of metabolism-related gene sets from Molecular Signatures Database (MSigDB). After integrated analysis, a risk score model based on the three most efficient prognostic DEMRGs, including Recombinant Phosphofructokinase Platelet (PFKP), phosphodiesterase 2A (PDE2A) and UDP-glucuronosyltransferase 1A5 (UGT1A5) was constructed and HCC patients were divided into high-risk and low-risk groups. Significant differences were found in pathway enrichment, immune cell infiltration patterns, and gene alterations between the two groups. High-risk group patients tended to have worse clinical outcomes and were more likely to respond to immunotherapy. A stemness-metabolism-related model composed of gender, age, the risk score model and tumor-node-metastasis (TNM) staging was generated and showed great discrimination and strong ability in predicting HCC prognosis and immunotherapy response.

## Introduction

Representing a globally major reason for death related to cancer, hepatocellular carcinoma (HCC) has a five-year overall survival (OS) rate of only 18% and is rising in incidence (1, 2). Poor survival is attributed to recurrence, relapse and treatment resistance. Specifically, 50-70% of patients with HCC experience a resurgence within five years after loco-regional therapy and up to 70% of relapsed patients recur within two years (3, 4). Treatment resistance is another common phenomenon in HCC. Immunotherapy (5) and sorafenib (6) have an objective response rate of approximately 15% and 9%, respectively, which are relatively low compared with other tumors. The efficacy is below expectation and high heterogeneity might be one of the reasons. Well-documented evidence has linked the heterogeneity of HCC with different clinical outcomes and diverse levels of sensitivity to treatment (7–9). Nevertheless, no classification accurately sub-classifying HCC patients, predicting prognosis and guiding treatment has been widely recognized in clinical practice up to now. Therefore, it is crucial to find a way to clearly distinguish HCC and elucidate biological and clinical characteristics simultaneously.

Recently, the theory of cancer stem cells (CSCs) has been generally accepted, providing new insights into cancer development and treatments. CSCs are a subgroup of tumor cells which have the potential to renew themselves and differentiate (10). A number of studies have already demonstrated the vital role of CSCs in HCC. Despite being small in number, CSCs are easily spread to distant organs, leading to HCC progression, recurrence (11) and metastasis (12). They are also implicated in therapy resistance, including sorafenib resistance (13–16), TACE refractoriness (17) and immunotherapy resistance (18). Moreover, CSCs could enhance immune evasion and induce an immunosuppressive microenvironment via the up-regulation of inhibitory molecules and the low expression of stimulatory molecules (19). They also attenuate the function of tumor-infiltrating lymphocytes by decreasing the expression of programmed cell death-ligand 1 (PD-L1), which is associated with immunotherapy response as well (20) (21). Several HCC CSC markers have been identified, including cluster of differentiation 90 (CD90), CD24, CD47, CD13, CD133, CD44, intercellular cell adhesion molecule-1 (ICAM1), epithelial cell adhesion molecule (EpCAM), leucine-rich repeat-containing receptor (LGR5), etc. (21). All the identified markers are separately reported to be tightly correlated with more aggressive HCC subtypes and worse outcomes (21). A few previous studies attempted to use CSC markers and gene signatures linked to CSC markers to identify HCC subtypes and predict HCC survival (22, 23). However, the results were unsatisfactory due to the heterogeneity of CSCs, and large-scale analysis is still needed to conclude how CSCs contribute to the prognosis of individual patients. It is necessary to conduct more research since the underlying mechanisms of CSCs remain unclear. A new stemness index, mRNAsi, was invented for the quantification of stemness on the basis of gene expression profiles (GEPs) (10). Ranging from zero (low) to one (high), the mRNAsi value was positively correlated with the similarity between tumor and stem cells. More than that, the mRNAsi value provided a new way of revealing the mechanism of CSCs in HCC, which was used as well during analysis in this article.

It has been universally accepted that energy metabolism reprogramming is a new emerging hallmark for cancer (24). The liver, which is an essential organ of metabolism, is vital in glucose and lipid homeostasis and responsible for more than 80% of protein synthesis. Diagnosed in the majority of HCC patients (68.4%) (25), metabolic associated fatty liver disease (MAFLD) has risen as one of the leading etiologies for HCC and received heated attention from researchers recently. Mounting research has shown that fatty acids, glucose, glutamine and amino acid metabolism pathways experience significant alternations in HCC owing to the energy demand for rapid cell multiplication. Glycolysis, glutamine catabolism as well as fatty acid synthesis and oxidation are enhanced, and the key enzymes included are highly expressed and related to poor clinical outcomes (26–29). Metabolic reprogramming is extremely deeply involved in the maintenance of CSCs compared with that of normal HCC cells. CSCs could preferentially survive a more restricted glucose supply by the increased expression of glucose transporter isoforms 1 (GLUT1) and GLUT3 (30). Stearoyl-coenzyme A desaturase 1 (SCD1) is an enzyme that catalyzes the desaturation of lipids, experiences a particular elevation in EpCAM^+^ alpha-fetoprotein (AFP)^+^ HCC and contributes to sorafenib resistance (31–33). CD13^+^CD133^+^HCC CSCs are deficient in Xanthine dehydrogenase/oxidase, an enzyme catalyzing purine catabolism (34). It is well-known that no studies have combined metabolism alterations with stemness features for the prediction of prognosis and immunotherapy response in HCC to date.

In this study, therefore, the transcriptomic data of patients with HCC were included to verify the following hypothesis: Stemness features were closely correlated with metabolism alterations in HCC patients, and the classification of patients based on a novel stemness-metabolism-related model could predict the clinical outcomes and immunotherapy response of HCC patients. Stemness features and mRNAsi were calculated by the OCLR algorithm. Metabolism-related gene sets were downloaded from MSigDB. Differential and weighted gene co-expression network analyses (WGCNA), univariate cox and least absolute shrinkage and selection operator (LASSO) regressions were performed and 3 most efficient prognostic DEMRGs: PFKP, PDE2A and UGT1A5 were identified. A risk score model was established and CIBERSORT, functional enrichment and copy number variation (CNV) analyses, estimation of stromal and immune cells in malignant tumor tissues using expression data (ESTIMATE), and Tumor immune dysfunction and exclusion (TIDE) were all explored between subgroups. A novel stemness-metabolism-related model was further constructed based on the risk score model and the areas under the receiver operating characteristic (ROC) curve (AUCs) of the novel model corresponding to the survival of one, three and five years were 0.744, 0.720 and 0.680 in training dataset, and the AUCs corresponding to the survival of one, three and five years in validation dataset were 0.633, 0.769 and 0.841, respectively. Collectively, a novel stemness-metabolism-related model was proposed and the vital role of metabolism reprogramming and CSCs in predicting the clinical outcomes of patients with HCC and potential immunotherapy response was highlighted. This study presented the first comprehensive analysis of genes genetically connected with the metabolic reprogramming and stemness features of HCC, providing several promising therapeutic targets.

## Materials and methods

### Data collection

The Cancer Genome Atlas Genomic Data Commons (TCGA GDC) website (https://portal.gdc.cancer.gov/) offered the RNA-sequencing (RNA-seq) profiles for the TCGA- liver hepatocellular carcinoma (LIHC) cohort. These profiles contain corresponding follow-up clinical information, such as gender, age, TNM staging, and survival status and time. After the exclusion of samples lacking complete clinical information, 363 samples were retained finally as a training dataset in total. **Table 1** displays detailed clinical information. Somatic mutation data were downloaded concurrently through Game Developers Conference (GDC), whose analysis and visualization were completed through the R package maftools (35). The validation dataset GSE76427 (36) (GPL10558) was downloaded from the Gene Expression Omnibus (GEO) database, and 115 samples were included after the exclusion of unqualified samples. The data were normalized using the R package limma (37).

**Table 1:** Baseline characteristics of TCGA-LIHC Patients from the TCGA Database.

Characteristic	levels	Overall
**n**		363
**Age, n (%)**	<60	165 (45.5%)
	>=60	198 (54.5%)
**Gender, n (%)**	female	118 (32.5%)
	male	245 (67.5%)
**Stage, n (%)**	not reported	9 (2.6%)
	stage I	170 (48.9%)
	stage II	84 (24.1%)
	stage III	81 (23.3%)
	stage IV	4 (1.1%)
**mRNAsi, median (IQR)**		0.38 (0.342, 0.422)

### Calculation of mRNAsi Based on GEPs

The previously reported one-class logistic regression (OCLR) algorithm (10) was adopted to predict and calculate mRNAsi based on the GEPs of pluripotent stem cells (PSCs). GEPs were acquired from the Progenitor Cell Biology Consortium (PCBC, https://progenitorcells.org/) and downloaded through the R synapser package. The mRNAsi value for every TCGA-LIHC sample is presented in **Table S1**.

### Analysis and screening of differentially expressed metabolism-related genes

Samples were stratified into low- and high-mRNAsi groups on the basis of the median mRNAsi value. Hallmark gene sets were downloaded from Molecular Signature Database (MSigDB) (38). A total of 2,752 metabolism-related genes were obtained after the removal of duplicated genes.

The R package DESeq2 (39) was used to differentially analyze the RNA-seq data from low- and high-mRNAsi groups and identify differentially expressed metabolism-related genes (DEMRGs) between the two groups. Adj. P value< 0.05 and | logFC | > 1 were regarded as the cutoff values for determining DEMRGs. The results are presented by the heatmap and volcano plot.

### Weighted gene co-expression network analysis

DEMRGs identified in the prior step were selected, and the R package weighted gene co-expression network analysis (WGCNA) (40) was utilized to perform WGCNA. Firstly, the coefficient of correlation between two random genes was calculated, whose weighted value was used for connecting the genes in the network submitting to scale-free networks. Next, the construction of a hierarchical clustering tree was based on inter-gene correlation coefficients. The clustering tree has a variety of branches representing different gene modules. Different colors stand for different modules. Then, a calculation was conducted for module significance (MS) which was used for measuring the correlations of mRNAsi values with different modules. Genes in every module were recorded and deemed as module eigengenes (MEs). Modules with the minimum and maximum MS values were perceived to be negative and positive modules, respectively. Modules of interest were chosen based on MS values, and MEs in those modules were considered highly correlated with mRNAsi values. Gene significance (GS) was utilized to measure the correlations between mRNAsi values and genes. Module membership (MM) was defined to be the association between an expression profile of DEMRGs and module genes.

### Construction of molecular subtypes based on DEMRGs

ConsensusClusterPlus (41), an R package, was applied to conduct unsupervised consensus clustering for the purpose of classifying LIHC patients into different subtypes in light of DEMRGs. The number of clusters was identified using consensus clustering which was performed with 1,000 iterations to make sure that the classification was stable. Ultimately, different patient subtypes were obtained.

### Establishment of a prognostic model based on DEMRGs

The identification of DEMRGs was based on differential expression analysis (DEA) and the WGCNA results. DEMRGs in low- and high-mRNAsi groups were analyzed first and the correlation was explored to check covariance, to analyze the expression of DEMRGs in LIHC. Significant DEMRGs were included in the model, and DEMRGs with prognostic significance were filtered by performing univariate Cox regression (P< 0.1). Next, the performance of least absolute shrinkage and selection operator (LASSO) regression reduced dimension and developed candidate prognostic DEMRGs, establishing the prognostic model. Computation was conducted for the risk score of HCC patients on the basis of this prognostic model in accordance with the normalized expression level and corresponding regression coefficients of each gene. The following formula was established. Patients were classified into low- and high-risk groups when the median risk score was set to be the cutoff value.


$$\text{risk}Score\;=\;\sum_{i}Coefficient\;\left(hub\;gene_{i}\right)*mRNA\;Expression\;$$

### Differential analysis of the prognostic risk score model

To identify metabolism- and stemness-related genes, the R package DESeq2 (39) was used for differentially analyzing the RNA-seq data from low- and high-risk groups in TCGA-LIHC. Adj. P Value< 0.05 and | logFC | > 1 were filter conditions. The results are presented by the heatmap and volcano plot.

### Functional enrichment and gene set enrichment analyses

Gene ontology (GO) (42), Kyoto Encyclopedia of Genes and Genomes (KEGG) (43) pathway analyses and other functional annotation and pathway enrichment analyses were carried out on DEMRGs by use of the R package clusterProfiler (44). The significance level was defined as false discovery rate (FDR)< 0.05.

Gene set enrichment analysis (GSEA) (45) was performed with the purpose of exploring the latent signaling pathways involved in GS between low- and high- risk groups in TCGA-LIHC. As a computing method of analyzing the statistical difference of a specific gene set between two biological states, GSEA is commonly applied to the estimation of changes in pathways and biological process activities in the samples of expression data sets. The download of the “c2.v7.2.symbols.gmt” gene set from MSigDB (46) was completed to perform GSEA and screen the metabolism-related results in the pathway. It was considered that pathways showed statistical significance with an FDR of less than 0.25. REACTOM (http://reactome.org/) and WikiPathways (https://www.wikipathways.org/index.php/WikiPathways) databases were employed to help demonstrate the results.

### Analysis of tumor immune cell infiltration

The use of estimation of stromal and immune cells in malignant tumor tissues using expression data (ESTIMATE) (47) evaluated the tumor microenvironment and quantified immune activity. ESTIMATE analysis predicted the abundance and tumor purity of intra-tumoral immune and stromal cells based on the GEPs of samples with HCC. A comparison was made between low- and high-risk groups in immune scores. Subsequently, CIBERSORT (48), a deconvolution algorithm on the basis of linear support vector regression, was employed for the further quantification and evaluation of 22 cell types related to immunity in a mixed population of infiltrating immune cells. The distribution of 22 immune infiltrating cells was presented using the R package ggplot2.

### Analysis of copy number variation

The copy number variation (CNV) data of TCGA-LIHC patients were obtained from TCGA and downloaded via the R package TCGAbiolinks (49). Genomic Identification of Significant Targets in Cancer (GISTIC) 2.0 (50) was harnessed to identify significant amplifications and deletions via GenePattern (51), and the results were visualized by the RCircos package in R. Default parameters were utilized except for confidential interval (CI)= 0.9 and no exclusion of X chromosome before analysis. The CNV burden was believed to be the total number of genes with CNVs in each sample.

### Immunotherapy response prediction

The tumor immune dysfunction and exclusion (TIDE) (52) (http://tide.dfci.harvard.edu) algorithm based on GEPs was used for predicting clinical response to the immune checkpoint blockade (ICB) of HCC patients. On the basis of the TIDE analysis results, immunotherapy-related factors were compared between low- and high-risk groups, including TIDE, CD8, CD274, etc.

### Prognostic value of the prognostic model based on mRNAsi-related metabolic genes

To demonstrate the personalized evaluation of risk scores combined with clinicopathological features for the prognosis of patients, the power in predicting OS was evaluated by conducting multivariate Cox regression analyses. Next, the risk score model in combination with clinicopathological features was chosen for inclusion in the model. Meanwhile, a nomogram was plotted for predicting the one-, three- and five-year OS of patients with HCC. Calibration curves were generated for assessing the performance of the nomogram by making a comparison between the predicted values and the observed actual rates of survival. The GSE76427 cohort was used as a validation dataset, and discrimination was evaluated through the receiver operating characteristic (ROC) curve.

### Statistical analysis

All statistical analyses were performed using R software 4.1.3. Independent Student’s and Wilcoxon tests for normally and non-normally distributed continuous data, respectively, were used for inter-group pairwise comparisons. Chi-square and Fisher’s exact tests were conducted to test the differences between categorical data. Survival analysis was performed using the R package survival. Differences in prognosis between the two patient groups were compared by conducting Kaplan-Meier (KM) curve analysis and log rank test. Time-dependent ROC curves were plotted, and prediction accuracy was evaluated by calculating the areas under the ROC curve (AUCs) (53). Univariate and multivariate analyses were both performed by means of Cox regression models to assess the performance of risk signature in predicting independent prognosis. It was deemed that a two-tailed P-value of below 0.05 showed statistical significance.

## Results

### Screening of mRNAsi-related metabolic genes and their expression profiles

**Figure 1:**
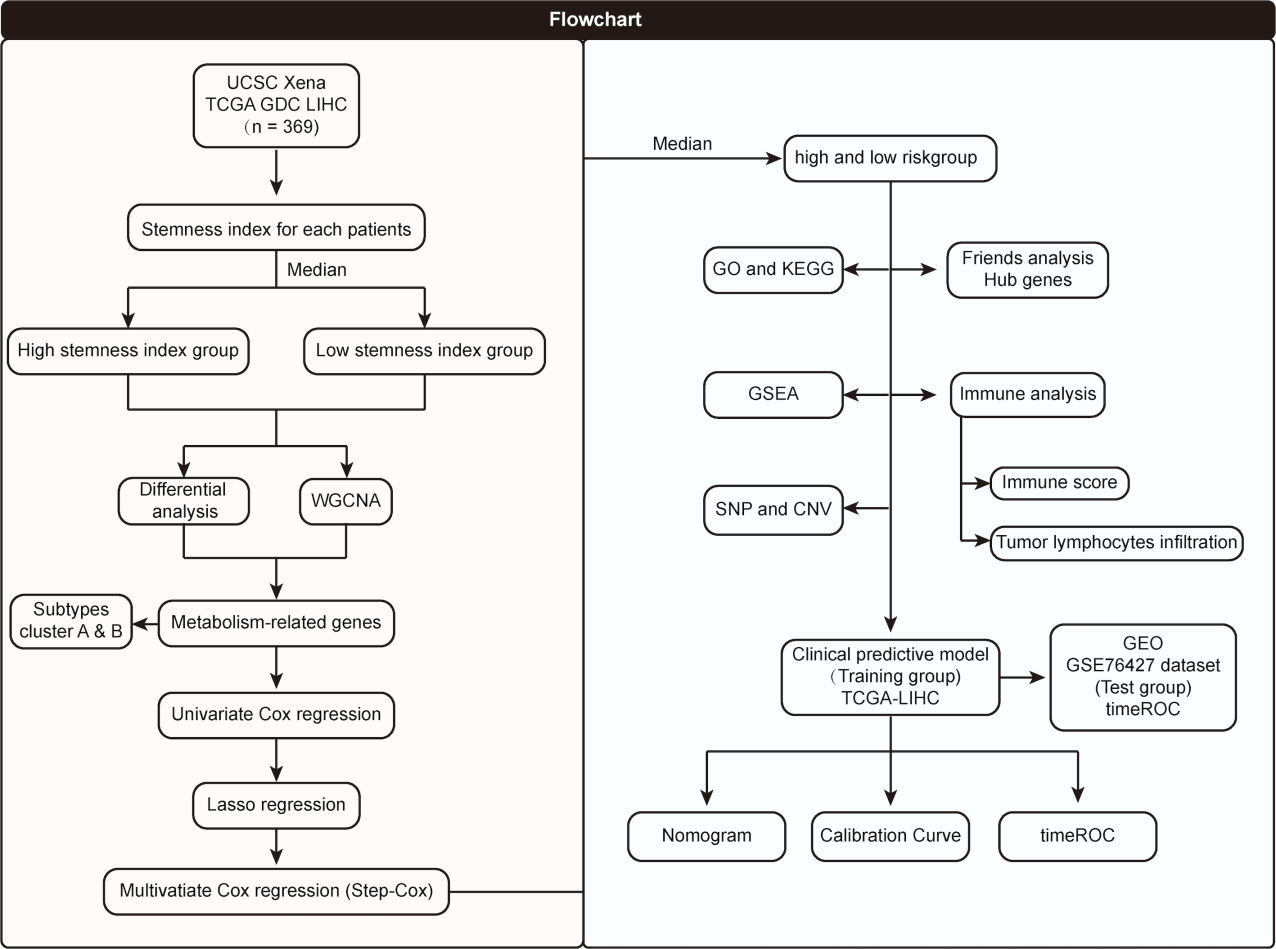
Flowchart of the entire research work.

**Figure 2:**
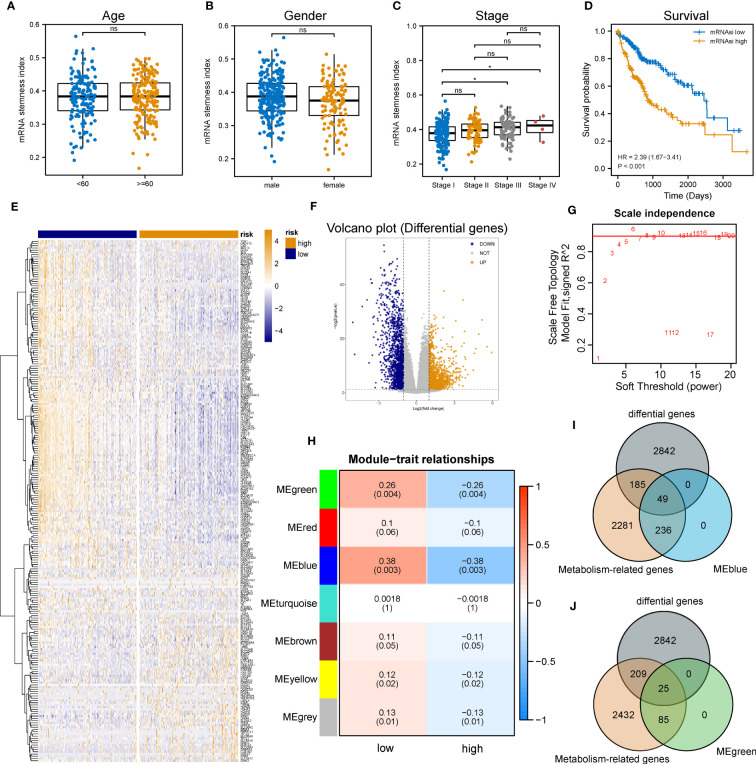
Identification of metabolic genes related to mRNAsi in patients with LIHC. **(A-C)** Boxplot of the associations of mRNAsi values with gender, age and stage, respectively. **(D)** Survival status between subgroups with low and high mRNAsi (Log-rank P< 0.001). **(E, F)** Heatmap and volcano plot of DEG expression in subgroups with low and high mRNAsi. **(G)** Sets of genes screened by WGCNA for the correlation with mRNAsi phenotypes. **(H)** Heatmap of associations and significant differences between a variety of mRNAsi scores and gene modules, where P values were shown in parentheses. **(I, J)** Venn diagrams of the intersection with DEGs, MEblue and MEgreen (ns and * represent P>0.05 and P<0.05, respectively).

The flowchart of the entire research work is presented in **Figure 1**. The OCLR algorithm was used to define mRNAsi. Stemness indices were presented for every patient in TCGA-LIHC and ranked from low to high in accordance with mRNAsi values to investigate the associations between clinicopathological characteristics and mRNAsi. No significant association was observed between mRNAsi and HCC patients’ age or gender (**Figures 2A, B**), but mRNAsi values differed between clinical stages. For instance, stage I patients exhibited significantly lower mRNAsi compared with stage III or IV ones (**Figure 2C**). Concurrently, survival analysis indicated the usually worse outcomes of patients with higher mRNAsi (Log-rank P< 0.001, **Figure 2D**).

Considering the impact of mRNAsi on the metabolism progression of HCC, patients with TCGA-LIHC were stratified into low- and high- groups according to the median stemness index value. In the meantime, DEA was performed between both groups. Beyond that, 3,076 differentially expressed genes (DEGs) including 1,536 down-regulated and 1,540 up-regulated genes were identified. The top 500 DEGs were visualized by a heatmap (**Figure 2E**), and all DEGs were drawn as a volcano map (**Figure 2F**). WGCNA was performed to further group co-expressing genes into a variety of modules for the identification of key mRNAsi-related metabolic genes. After analysis, it was found that the optimal soft threshold was 5 (**Figure 2G**), and seven effective modules were obtained (**Figure 2H**). The significance values of seven modules through the connection between mRNAsi and each module and the two most correlated modules were found, namely MEblue and MEgreen (**Figures 2I, J**). Thus, the intersection genes of the MEblue and MEgreen modules with the DEGs identified before were selected, and 74 DEMRGs were obtained.

**Figure 3:**
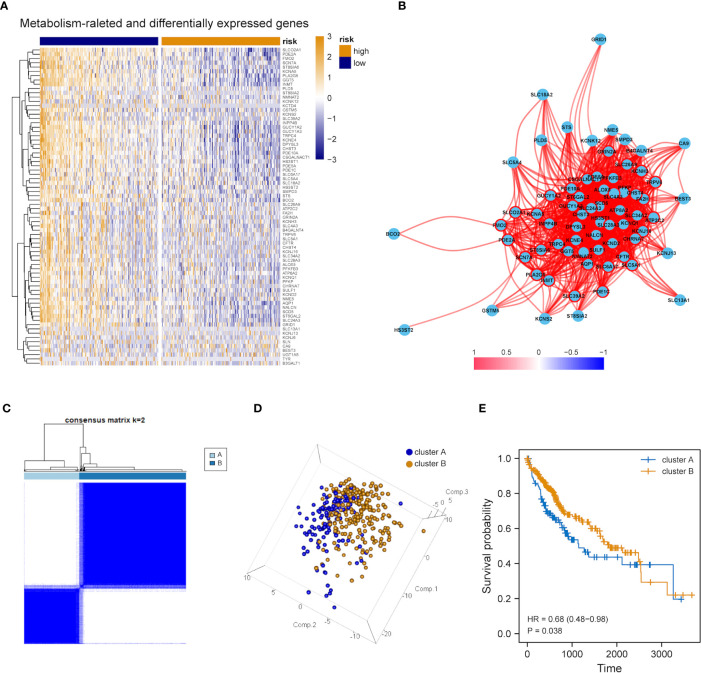
Overall expression of DEMRGs in LIHC patients. **(A)** Heatmap of 74 DEMRGs in normal and LIHC tissues. Yellow represents high gene expression, blue represents low gene expression, darker yellow represents higher gene expression level, and darker blue represents lower gene expression level. **(B)** Correlation map of 74 DEMRGs. Red represents a positive correlation, blue represents a negative correlation, darker red represents a higher positive correlation, and darker blue represents a higher negative correlation. **(C)** Heatmap of the clustering analysis results of DEMRGs, k=2 sample clustering. Darker blue represents the higher expression level of characteristic genes, conversely, lighter blue represents the lower expression level of genes. **(D)** Principal component analysis of two subgroups shown in a three-dimensional model. **(E)** Prognostic analysis of the subgroups.

The expression tendency of those 74 DEMRGs was further mapped to find the down-regulation of most candidate genes in the group with high mRNAsi (compared with the group with low mRNAsi) (**Figure 3A**). Then, co-expression network analysis was performed. It was noticed that candidate genes were strongly correlated in their modules, consistent with previous WGCNA results (**Figure 3B**). Based on the selected DEMRGs, LIHC patients could be divided into two clusters. It was found that survival probability exhibited a significant difference between clusters A and B, indicating that DEMRGs could serve as good prognostic factors for LIHC (**Figures 3C–E**).

### Establishment of a DEMRGs-based prognostic risk score model

**Figure 4:**
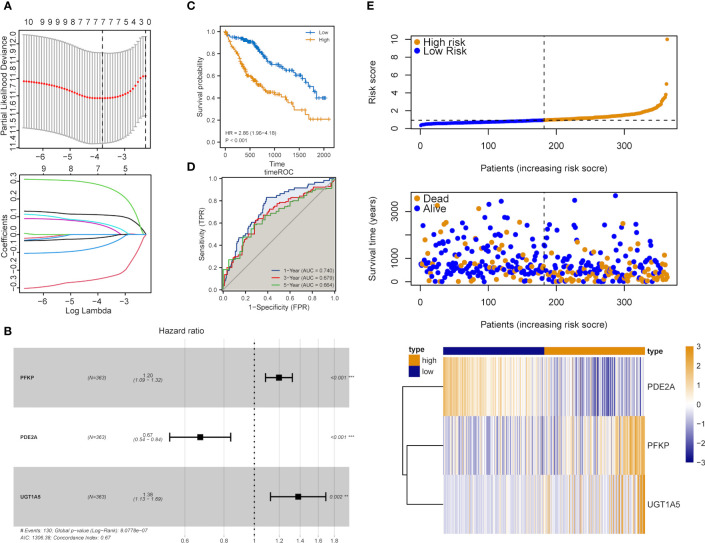
Construction of the metabolic risk score model related to mRNAsi. **(A)** LASSO regression analysis results and the optimal lambda value corresponding to seven variables. **(B)** Multivariate cox regression analysis results and three identified genes as independent prognostic factors. **(C)** K-M curve of risk scores for the OS of patients with HCC. **(D)** ROC curve analysis of risk scores dependent on time. **(E)** Distribution of risk scores, the survival of patients and the heatmap of expression of characteristic genes in LIHC patients.

The influence of DEMRGs on LIHC prognosis was quantified via the establishment of a prognostic risk score model. Firstly, univariate Cox regression was performed, and 11 qualified genes were filtered. Secondly, LASSO and multivariate Cox regressions were conducted. Finally, three genes, including Recombinant Phosphofructokinase, Platelet (PFKP), phosphodiesterase 2A (PDE2A) and UDP-glucuronosyltransferase 1-5 (UGT1A5), were identified as the most efficient prognostic genes (**Figures 4A, B**).

Subsequently, the above-mentioned three genes were applied to build a multi-gene signature for predicting the survival of HCC. Coefficients acquired from multivariate Cox regression were used for calculating the risk score of each patient with HCC. Below was the risk score formula:


riskScore=PKFP*(0.18)+PDE2A*(−0.40)+UGT1A5*(0.32)

K-M analysis indicated the poorer OS of patients in the high-risk group (Log-rank P<0.001, **Figure 4C**). The ROC curve dependent on time demonstrated that the three-gene signature showed the good predictive value and the risk scores corresponding to the survival of one, three and five years had an AUC of 0.740, 0.679 and 0.664, respectively (**Figure 4D**). The distribution of risk scores, survival status as well as three-gene expression pattern are illustrated in **Figure 4E**.

### DEA between low- and high-risk groups

**Figure 5:**
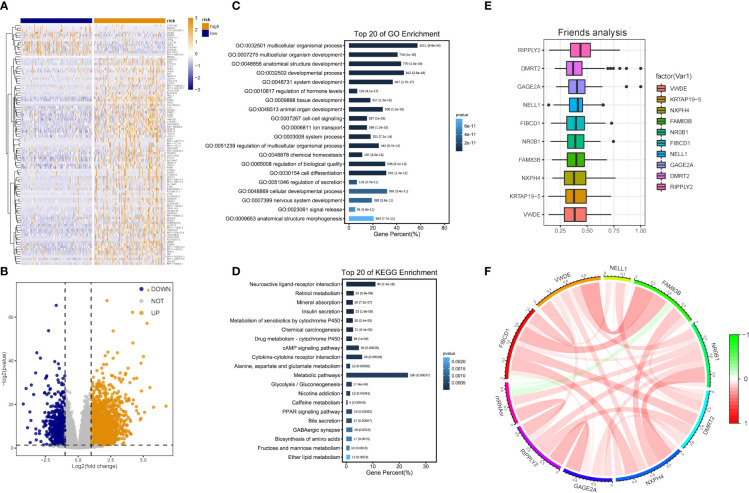
DEG and functional enrichment analyses for the metabolic risk model related to mRNAsi. **(A, B)** Volcano map and heatmap of expression of DEGs between low- and high-risk LIHC patients in TCGA and GEO datasets. **(C)** GO analysis indicated a close association between significant DEGs and multicellular organismal, developmental and biological processes. **(D)** KEGG analysis showed the involvement of DEGs in metabolic and cAMP signaling pathways. **(E)** Identification of top 10 hub genes from the analysis of Friends. **(F)** Correlation analysis of hub genes.

Patients with DEA were categorized into low- and high-risk groups following the median score value to further analyze the association between the risk model and HCC development. DEA between both groups was performed, and 3,806 DEGs including 777 down-regulated and 3,029 up-regulated genes were identified (**Figures 5A, B**).

Functional enrichment analyses were performed on 3,806 DEGs. The GO results revealed that DEGs were primarily abundant in multicellular organismal and developmental processes, multicellular organism development and other biological processes (BPs) (**Figure 5C**). The KEGG analysis results indicated that DEGs mainly participated in metabolic, neuroactive ligand-receptor interaction and cyclic adenosine monophosphate (cAMP) signaling pathways, etc. (**Figure 5D**). It was discovered that several results were correlated with the progression of metabolism, such as amino acid, caffeine, retinol and ether lipid metabolism. The detailed results are supplied in **Tables 2**, **3**.

**Table 2:** GO analysis of differentially expressed genes.

Class	ID	Description	Qvalue	number
BP	GO:0032501	multicellular organismal process	8.89E-30	1011
BP	GO:0007275	multicellular organism development	4.49E-16	719
BP	GO:0048856	anatomical structure development	5.72E-16	770
BP	GO:0032502	developmental process	5.72E-16	812
BP	GO:0048731	system development	3.07E-14	647
BP	GO:0010817	regulation of hormone levels	6.05E-14	122
BP	GO:0009888	tissue development	1.51E-13	317
BP	GO:0048513	animal organ development	1.63E-13	500
BP	GO:0007267	cell-cell signaling	1.02E-12	267
BP	GO:0006811	ion transport	1.08E-12	266
BP	GO:0003008	system process	5.79E-11	321
BP	GO:0051239	regulation of multicellular organismal process	7.24E-11	441
BP	GO:0048878	chemical homeostasis	1.80E-10	197
BP	GO:0065008	regulation of biological quality	5.18E-10	536
BP	GO:0030154	cell differentiation	8.31E-10	551
BP	GO:0051046	regulation of secretion	1.50E-08	118
BP	GO:0048869	cellular developmental process	1.78E-08	562
BP	GO:0007399	nervous system development	1.78E-08	338
BP	GO:0023061	signal release	2.62E-08	91
BP	GO:0009653	anatomical structure morphogenesis	3.46E-08	364
CC	GO:0031226	intrinsic component of plasma membrane	4.90E-21	301
CC	GO:0005887	integral component of plasma membrane	2.24E-18	281
CC	GO:0044459	plasma membrane part	1.99E-17	430
CC	GO:0005576	extracellular region	1.99E-17	593
CC	GO:0031224	intrinsic component of membrane	8.31E-12	730
CC	GO:0098590	plasma membrane region	2.92E-10	196
CC	GO:0005886	plasma membrane	5.87E-10	686
CC	GO:0005615	extracellular space	5.87E-10	444
CC	GO:0044421	extracellular region part	6.93E-10	469
CC	GO:0071944	cell periphery	9.38E-10	697
CC	GO:0016021	integral component of membrane	2.43E-09	696
CC	GO:0044425	membrane part	5.36E-07	827
CC	GO:0031012	extracellular matrix	2.91E-06	97
CC	GO:0097458	neuron part	3.02E-06	250
CC	GO:1902495	transmembrane transporter complex	1.15E-05	63
CC	GO:0097060	synaptic membrane	1.26E-05	71
CC	GO:0016324	apical plasma membrane	1.43E-05	68
CC	GO:1990351	transporter complex	2.29E-05	64
CC	GO:0045177	apical part of cell	2.86E-05	76
CC	GO:0016323	basolateral plasma membrane	5.67E-05	50
MF	GO:0048018	receptor ligand activity	2.51E-10	103
MF	GO:0030545	receptor regulator activity	4.69E-10	109
MF	GO:0022857	transmembrane transporter activity	2.19E-08	173
MF	GO:0015267	channel activity	2.19E-08	92
MF	GO:0022803	passive transmembrane transporter activity	2.19E-08	92
MF	GO:0015075	ion transmembrane transporter activity	3.27E-08	146
MF	GO:0022838	substrate-specific channel activity	3.53E-08	85
MF	GO:0015318	inorganic molecular entity transmembrane transporter activity	3.53E-08	138
MF	GO:0005215	transporter activity	9.56E-08	182
MF	GO:0005216	ion channel activity	1.70E-07	81
MF	GO:0005179	hormone activity	5.87E-07	36
MF	GO:0022836	gated channel activity	9.28E-07	69
MF	GO:0022839	ion gated channel activity	1.59E-06	67
MF	GO:0005102	signaling receptor binding	1.71E-06	232
MF	GO:0005261	cation channel activity	4.22E-06	64
MF	GO:0020037	heme binding	4.41E-06	37
MF	GO:0046873	metal ion transmembrane transporter activity	7.29E-06	78
MF	GO:0046906	tetrapyrrole binding	8.24E-06	38
MF	GO:0016712	oxidoreductase activity, acting on paired donors, with incorporation or reduction of molecular oxygen, reduced flavin or flavoprotein as one donor, and incorporation of one atom of oxygen	8.29E-06	16
MF	GO:0008395	steroid hydroxylase activity	1.26E-05	16

**Table 3:** KEGG analysis of significantly differentially expressed metabolism-related genes.

class	ID	Description	Qvalue	num
Metabolism	ko00830	Retinol metabolism	8.82E-06	23
Metabolism	ko00980	Metabolism of xenobiotics by cytochrome P450	1.56E-03	20
Metabolism	ko00982	Drug metabolism - cytochrome P450	4.73E-03	18
Metabolism	ko00250	Alanine, aspartate and glutamate metabolism	9.81E-03	12
Metabolism	ko01100	Metabolic pathways	9.81E-03	190
Metabolism	ko00010	Glycolysis / Gluconeogenesis	9.81E-03	17
Metabolism	ko00232	Caffeine metabolism	9.81E-03	4
Metabolism	ko01230	Biosynthesis of amino acids	2.53E-02	17
Metabolism	ko00051	Fructose and mannose metabolism	2.53E-02	10
Metabolism	ko00565	Ether lipid metabolism	3.64E-02	12
Metabolism	ko00590	Arachidonic acid metabolism	4.21E-02	15
Metabolism	ko00410	beta-Alanine metabolism	4.21E-02	9
Metabolism	ko00052	Galactose metabolism	4.94E-02	9
Metabolism	ko00140	Steroid hormone biosynthesis	4.94E-02	14
Metabolism	ko00910	Nitrogen metabolism	5.46E-02	6
Metabolism	ko00340	Histidine metabolism	7.94E-02	7
Metabolism	ko01200	Carbon metabolism	8.88E-02	21
Metabolism	ko00030	Pentose phosphate pathway	8.88E-02	8
Metabolism	ko00601	Glycosphingolipid biosynthesis - lacto and neolacto series	1.58E-01	7
Metabolism	ko00040	Pentose and glucuronate interconversions	1.70E-01	8
Metabolism	ko00120	Primary bile acid biosynthesis	1.79E-01	5
Metabolism	ko00591	Linoleic acid metabolism	1.99E-01	7
Metabolism	ko00360	Phenylalanine metabolism	2.08E-01	5
Metabolism	ko00512	Mucin type O-glycan biosynthesis	2.51E-01	7
Metabolism	ko00290	Valine, leucine and isoleucine biosynthesis	3.07E-01	2
Metabolism	ko00650	Butanoate metabolism	3.07E-01	6
Metabolism	ko00790	Folate biosynthesis	3.07E-01	6
Metabolism	ko00430	Taurine and hypotaurine metabolism	3.07E-01	4
Metabolism	ko00730	Thiamine metabolism	3.51E-01	4
Metabolism	ko00500	Starch and sucrose metabolism	3.51E-01	7
Metabolism	ko00380	Tryptophan metabolism	3.66E-01	8
Metabolism	ko00524	Neomycin, kanamycin and gentamicin biosynthesis	3.89E-01	2
Metabolism	ko00350	Tyrosine metabolism	3.97E-01	7
Metabolism	ko00983	Drug metabolism - other enzymes	4.17E-01	12
Metabolism	ko00472	D-Arginine and D-ornithine metabolism	4.26E-01	1
Metabolism	ko00660	C5-Branched dibasic acid metabolism	4.26E-01	1
Metabolism	ko00600	Sphingolipid metabolism	4.43E-01	8
Metabolism	ko00400	Phenylalanine, tyrosine and tryptophan biosynthesis	4.54E-01	2
Metabolism	ko01040	Biosynthesis of unsaturated fatty acids	4.74E-01	5
Metabolism	ko00260	Glycine, serine and threonine metabolism	4.87E-01	7
Metabolism	ko00561	Glycerolipid metabolism	6.23E-01	9
Metabolism	ko00330	Arginine and proline metabolism	7.54E-01	7
Metabolism	ko00450	Selenocompound metabolism	8.26E-01	3
Metabolism	ko01210	2-Oxocarboxylic acid metabolism	8.26E-01	3
Metabolism	ko00564	Glycerophospholipid metabolism	8.26E-01	12
Metabolism	ko00053	Ascorbate and aldarate metabolism	8.26E-01	4
Metabolism	ko00062	Fatty acid elongation	8.26E-01	4
Metabolism	ko00071	Fatty acid degradation	8.26E-01	6
Metabolism	ko00130	Ubiquinone and other terpenoid-quinone biosynthesis	9.05E-01	2
Metabolism	ko01212	Fatty acid metabolism	9.54E-01	7
Metabolism	ko00280	Valine, leucine and isoleucine degradation	9.60E-01	6
Metabolism	ko00270	Cysteine and methionine metabolism	1.00E+00	6
Metabolism	ko00220	Arginine biosynthesis	1.00E+00	3
Metabolism	ko00860	Porphyrin and chlorophyll metabolism	1.00E+00	5
Metabolism	ko00471	D-Glutamine and D-glutamate metabolism	1.00E+00	1
Metabolism	ko00603	Glycosphingolipid biosynthesis - globo and isoglobo series	1.00E+00	2
Metabolism	ko00534	Glycosaminoglycan biosynthesis - heparan sulfate / heparin	1.00E+00	3
Metabolism	ko00592	alpha-Linolenic acid metabolism	1.00E+00	3
Metabolism	ko00514	Other types of O-glycan biosynthesis	1.00E+00	5
Metabolism	ko00480	Glutathione metabolism	1.00E+00	6
Metabolism	ko00770	Pantothenate and CoA biosynthesis	1.00E+00	2
Metabolism	ko00520	Amino sugar and nucleotide sugar metabolism	1.00E+00	5
Metabolism	ko00620	Pyruvate metabolism	1.00E+00	4
Metabolism	ko00020	Citrate cycle (TCA cycle)	1.00E+00	3
Metabolism	ko00072	Synthesis and degradation of ketone bodies	1.00E+00	1
Metabolism	ko00100	Steroid biosynthesis	1.00E+00	2
Metabolism	ko00640	Propanoate metabolism	1.00E+00	3
Metabolism	ko00760	Nicotinate and nicotinamide metabolism	1.00E+00	3
Metabolism	ko00533	Glycosaminoglycan biosynthesis - keratan sulfate	1.00E+00	1
Metabolism	ko00310	Lysine degradation	1.00E+00	5
Metabolism	ko00513	Various types of N-glycan biosynthesis	1.00E+00	3
Metabolism	ko00604	Glycosphingolipid biosynthesis - ganglio series	1.00E+00	1
Metabolism	ko00230	Purine metabolism	1.00E+00	10
Metabolism	ko00531	Glycosaminoglycan degradation	1.00E+00	1
Metabolism	ko00532	Glycosaminoglycan biosynthesis - chondroitin sulfate / dermatan sulfate	1.00E+00	1
Metabolism	ko00515	Mannose type O-glycan biosyntheis	1.00E+00	1
Metabolism	ko00563	Glycosylphosphatidylinositol(GPI)-anchor biosynthesis	1.00E+00	1
Metabolism	ko00240	Pyrimidine metabolism	1.00E+00	3
Metabolism	ko00562	Inositol phosphate metabolism	1.00E+00	4
Metabolism	ko00630	Glyoxylate and dicarboxylate metabolism	1.00E+00	1
Metabolism	ko00510	N-Glycan biosynthesis	1.00E+00	1
Metabolism	ko00190	Oxidative phosphorylation	1.00E+00	5

The analysis results of Friends also implied that recombinant human protein ripply2 (RIPPLY2, DMRT2), G antigen 2A (GAGE2A) and 10 other genes might have an important influence. This meant that they could be hub genes as well, which were mostly up-regulated in functional analyses consistently (**Figures 5E, F**).

**Figure 6:**
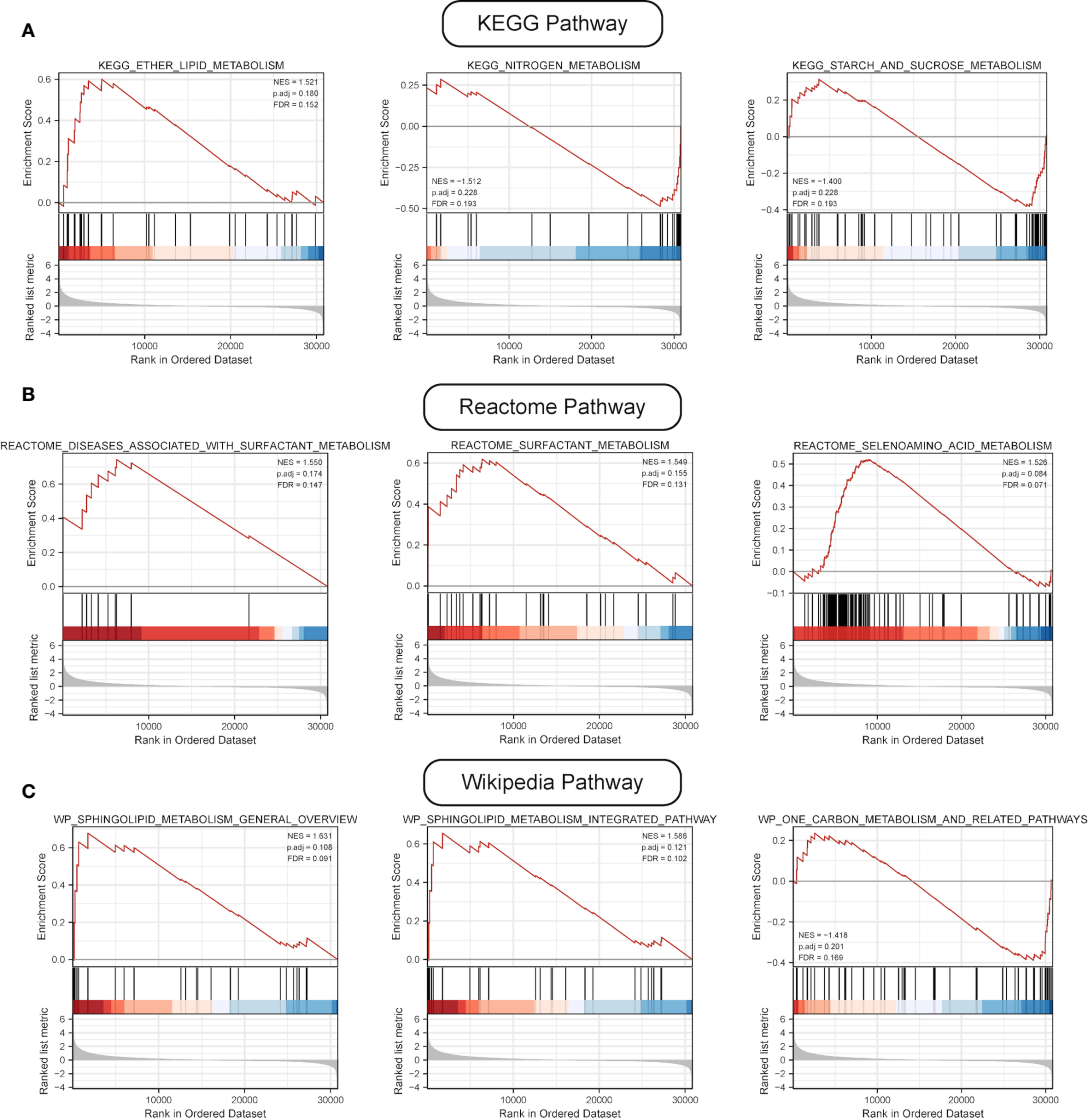
Results of GSEA. **(A)** Results of top three pathways of KEGG-based metabolic pathway analysis. **(B)** Results of top three pathways of Reactome-based metabolic pathway analysis. **(C)** Results of top three pathways of WP-based metabolic pathway analysis.

GSEA was performed, and the results of WikiPathways, KEGG and REACTOM databases were summarized according to the C2 pathway. The results demonstrated that the high-risk group mainly showed enriched ether lipid metabolism while the low-risk one mainly exhibited enriched starch and sucrose and nitrogen metabolism (**Figure 6A**). In the REACTOM pathway database, the high-risk group demonstrated significantly enriched disease associated with surfactant metabolism, and surfactant and selenoamino acid metabolism (**Figure 6B**). In the WIKIPEDIA pathway database, the high-risk group witnessed the up-regulation of the general overview and integrated pathway of sphingolipid metabolism, whereas the low-risk one went through the down-regulation of one-carbon metabolism and related pathways (**Figure 6C**). The detailed GSEA results are shown in **Table 4**.

**Table 4:** GSEA analysis results of metabolism-related genes.

ID	setSize	NES	pvalue	FDR
KEGG_ALANINE_ASPARTATE_AND_GLUTAMATE_METABOLISM	30	-1.64	3.01E-02	1.64E-01
KEGG_ARGININE_AND_PROLINE_METABOLISM	53	-1.92	1.68E-02	1.20E-01
KEGG_BETA_ALANINE_METABOLISM	22	-1.82	9.52E-03	8.95E-02
KEGG_BUTANOATE_METABOLISM	34	-1.75	6.71E-03	7.70E-02
KEGG_CYSTEINE_AND_METHIONINE_METABOLISM	34	-1.89	6.71E-03	7.70E-02
KEGG_DRUG_METABOLISM_CYTOCHROME_P450	71	-2.30	1.11E-02	9.40E-02
KEGG_DRUG_METABOLISM_OTHER_ENZYMES	51	-1.90	8.13E-03	8.29E-02
KEGG_ETHER_LIPID_METABOLISM	30	1.52	2.63E-02	1.52E-01
KEGG_FATTY_ACID_METABOLISM	42	-3.00	6.99E-03	7.70E-02
KEGG_GLYCINE_SERINE_AND_THREONINE_METABOLISM	31	-2.61	6.13E-03	7.70E-02
KEGG_GLYOXYLATE_AND_DICARBOXYLATE_METABOLISM	16	-1.70	3.17E-02	1.67E-01
KEGG_HISTIDINE_METABOLISM	28	-1.91	5.56E-03	7.70E-02
KEGG_LINOLEIC_ACID_METABOLISM	29	-1.64	2.34E-02	1.44E-01
KEGG_METABOLISM_OF_XENOBIOTICS_BY_CYTOCHROME_P450	69	-1.85	1.08E-02	9.32E-02
KEGG_NITROGEN_METABOLISM	23	-1.51	4.33E-02	1.93E-01
KEGG_PROPANOATE_METABOLISM	32	-2.34	6.37E-03	7.70E-02
KEGG_PYRUVATE_METABOLISM	40	-1.53	2.72E-02	1.55E-01
KEGG_RETINOL_METABOLISM	64	-2.31	9.62E-03	8.95E-02
KEGG_STARCH_AND_SUCROSE_METABOLISM	52	-1.40	4.27E-02	1.93E-01
KEGG_TRYPTOPHAN_METABOLISM	39	-2.30	6.45E-03	7.70E-02
KEGG_TYROSINE_METABOLISM	40	-1.79	6.80E-03	7.70E-02
REACTOME_ABACAVIR_TRANSPORT_AND_METABOLISM	10	-2.07	3.44E-03	7.11E-02
REACTOME_ALPHA_LINOLENIC_OMEGA3_AND_LINOLEIC_OMEGA6_ACID_METABOLISM	13	-1.51	5.82E-02	2.34E-01
REACTOME_ARACHIDONIC_ACID_METABOLISM	58	-1.58	1.82E-02	1.26E-01
REACTOME_BILE_ACID_AND_BILE_SALT_METABOLISM	43	-2.25	7.30E-03	7.75E-02
REACTOME_BIOTIN_TRANSPORT_AND_METABOLISM	11	-1.86	2.03E-02	1.33E-01
REACTOME_CARNITINE_METABOLISM	14	-1.79	3.40E-02	1.71E-01
REACTOME_DEFECTS_IN_VITAMIN_AND_COFACTOR_METABOLISM	20	-1.62	2.82E-02	1.57E-01
REACTOME_DISEASES_ASSOCIATED_WITH_SURFACTANT_METABOLISM	10	1.55	2.39E-02	1.47E-01
REACTOME_DISEASES_OF_CARBOHYDRATE_METABOLISM	34	-1.61	2.01E-02	1.33E-01
REACTOME_FATTY_ACID_METABOLISM	169	-2.23	4.00E-02	1.87E-01
REACTOME_FOXO_MEDIATED_TRANSCRIPTION_OF_OXIDATIVE_STRESS_METABOLIC_AND_NEURONAL_GENES	30	-1.80	6.02E-03	7.70E-02
REACTOME_GLYCOGEN_METABOLISM	27	-1.71	1.06E-02	9.32E-02
REACTOME_GLYOXYLATE_METABOLISM_AND_GLYCINE_DEGRADATION	31	-2.33	6.13E-03	7.70E-02
REACTOME_KETONE_BODY_METABOLISM	10	-1.68	3.78E-02	1.83E-01
REACTOME_METABOLISM_OF_ANGIOTENSINOGEN_TO_ANGIOTENSINS	17	-2.20	4.13E-03	7.11E-02
REACTOME_METABOLISM_OF_PORPHYRINS	26	-1.71	1.09E-02	9.32E-02
REACTOME_METABOLISM_OF_STEROIDS	148	-1.60	3.13E-02	1.67E-01
REACTOME_PEROXISOMAL_LIPID_METABOLISM	29	-2.44	5.85E-03	7.70E-02
REACTOME_PYRUVATE_METABOLISM_AND_CITRIC_ACID_TCA_CYCLE	55	-1.49	1.83E-02	1.26E-01
REACTOME_REGULATION_OF_LIPID_METABOLISM_BY_PPARALPHA	118	-1.53	1.96E-02	1.31E-01
REACTOME_SELENOAMINO_ACID_METABOLISM	109	1.53	3.17E-03	7.11E-02
REACTOME_SPHINGOLIPID_METABOLISM	84	1.34	5.63E-02	2.29E-01
REACTOME_SULFUR_AMINO_ACID_METABOLISM	26	-2.56	5.43E-03	7.70E-02
REACTOME_SURFACTANT_METABOLISM	28	1.55	1.95E-02	1.31E-01
WP_AMINO_ACID_METABOLISM	85	-2.33	1.30E-02	1.03E-01
WP_EICOSANOID_METABOLISM_VIA_CYCLO_OXYGENASES_COX	29	-1.70	1.75E-02	1.24E-01
WP_EICOSANOID_METABOLISM_VIA_LIPO_OXYGENASES_LOX	29	-1.80	5.85E-03	7.70E-02
WP_ENERGY_METABOLISM	47	-1.43	4.03E-02	1.88E-01
WP_ESTROGEN_METABOLISM	18	-1.69	3.46E-02	1.72E-01
WP_FOLATE_METABOLISM	70	-1.87	1.08E-02	9.32E-02
WP_IRON_METABOLISM_IN_PLACENTA	12	-1.75	3.66E-02	1.78E-01
WP_METABOLIC_PATHWAY_OF_LDL_HDL_AND_TG_INCLUDING_DISEASES	16	-1.82	1.98E-02	1.31E-01
WP_METHIONINE_METABOLISM_LEADING_TO_SULPHUR_AMINO_ACIDS_AND_RELATED_DISORDERS	11	-2.30	3.39E-03	7.11E-02
WP_NAD_METABOLISM_SIRTUINS_AND_AGING	11	-1.60	6.10E-02	2.43E-01
WP_NUCLEAR_RECEPTORS_IN_LIPID_METABOLISM_AND_TOXICITY	35	-2.20	6.94E-03	7.70E-02
WP_ONE_CARBON_METABOLISM	30	-1.77	1.81E-02	1.26E-01
WP_ONE_CARBON_METABOLISM_AND_RELATED_PATHWAYS	50	-1.42	3.28E-02	1.69E-01
WP_SPHINGOLIPID_METABOLISM_GENERAL_OVERVIEW	22	1.63	1.01E-02	9.12E-02
WP_SPHINGOLIPID_METABOLISM_INTEGRATED_PATHWAY	23	1.59	1.26E-02	1.02E-01
WP_TAMOXIFEN_METABOLISM	21	-2.02	4.61E-03	7.17E-02
WP_TRANSSULFURATION_AND_ONE_CARBON_METABOLISM	30	-1.59	3.01E-02	1.64E-01
WP_TRYPTOPHAN_METABOLISM	41	-2.40	6.94E-03	7.70E-02
WP_UREA_CYCLE_AND_METABOLISM_OF_AMINO_GROUPS	20	-1.58	3.76E-02	1.82E-01
WP_VITAMIN_B12_METABOLISM	49	-1.78	1.60E-02	1.16E-01

### Correlations between the risk score model and patterns of tumor immune cell infiltration

**Figure 7:**
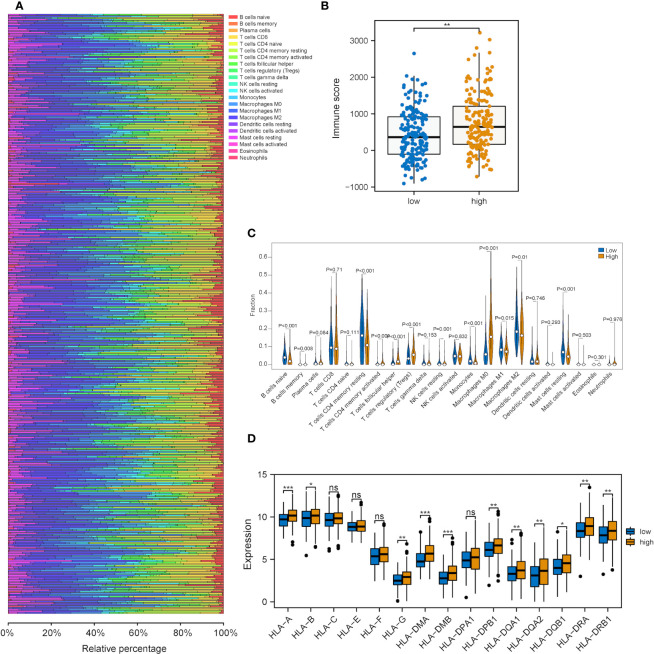
Correlation analysis between the metabolic risk score model related to mRNAsi and the level of immune infiltration. **(A, B)** Immune and stromal scores of the high-risk group experienced a significant increase in comparison with those of the low-risk group (immune and stromal scores: *P<*0.001). **(C)** Heatmap of the infiltration level of 22 immune cells in GEO and TCGA datasets. **(D)** Heatmap of the association between different immune cell infiltration levels. (ns, *, ** and *** represent P>0.05, P<0.05, P<0.01 and P<0.001, respectively).

Next, we explored the correlations between the risk score of the three-gene signature and the immune cell infiltration and tumor immune pattern of HCC. Overall immune infiltration is shown in **Figure 7A**. According to the ESTIMATE results, HCC patients in the high-risk group exhibited a statistical elevation in immune and stromal scores compared with those in the low-risk group (**Figure 7B**). Meanwhile, it was discovered that the infiltration levels of several immune cell types showed significant differences and highly-abundant regulatory T cells (Tregs) and macrophages (M0) in the high-risk group but highly-abundant naïve B and activated memory CD4+ T cells in the low-risk group (**Figure 7C**). Besides, low- and high-risk groups showed a significant difference in the expression of several human leukocyte antigen (HLA) family genes (**Figure 7D**).

### Correlations between the risk score model and genomic alternations

**Figure 8:**
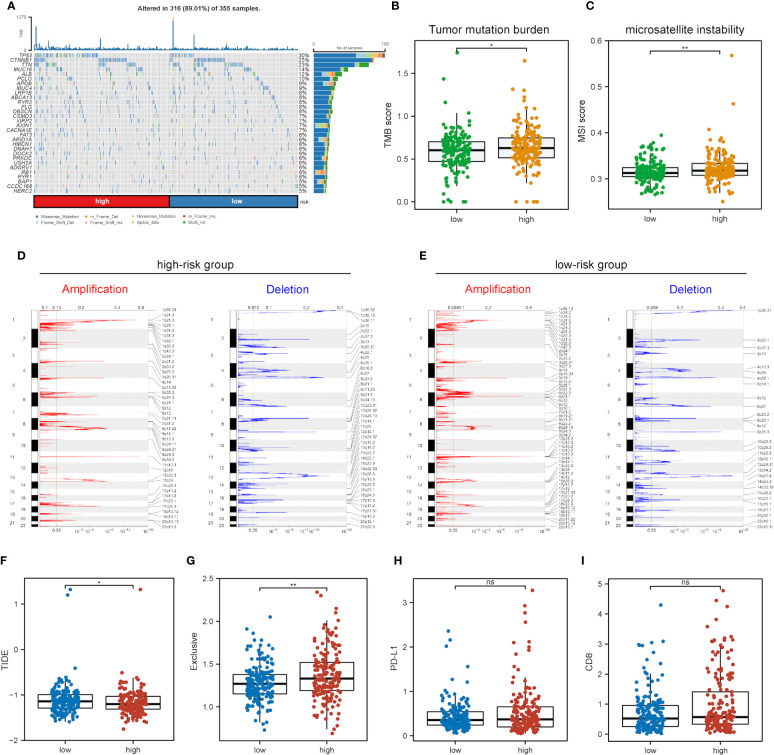
Impact of different mRNAsi-related metabolic risk subgroups on genetic variants and immunotherapy. **(A)** The waterfall plot shows the mutation profiles of commonly-seen tumorigenic driver genes of patients in low- and high-risk subgroups, with a variety of colors indicating different types of mutation and vignettes above the legend indicating mutational load. **(B, C)** Boxplots of differences in TMB and MSI levels between patients in low- and high-risk groups, respectively. **(D, E)** Comparison of the CNV levels of various genes between low- and high-risk groups. Red and blue indicated genes with significantly increased and decreased CNVs, respectively. **(F-I)** Differences in TIDE, Exclusive, PD-L1 and CD8 scores between low- and high-risk groups based on the TIDE database (ns, * and ** represent P>0.05, P<0.05 and P<0.01, respectively).

Further exploration was conducted into the correlations of risk scores with genomic alterations, including single nucleotide polymorphisms (SNPs), CNVs, etc. Somatic mutation analysis revealed that both low- and high-risk groups possessed their specific top mutant genes (**Figure 8A**). Patients in the high-risk group possessed high-level microsatellite instability (MSI) and tumor mutation burden (TMB), which indicated that they obtained more genomic alterations (**Figures 8B, C**). CNV analysis showed that HCC patients exhibited plenty of CNVs while patients in different groups contained different CNV patterns (**Figures 8D, E**).

As immunotherapy is playing an increasingly important role in tumor treatment, the TIDE algorithm was used for predicting the immunotherapy sensitivity of patients in both low- and high-risk groups. It can be seen from **Figure 8F** that TIDE scores were lower in the high-risk group than the low-risk one, indicating the possibly less sensitivity of patients in the high-risk group. Exclusive scores were utilized to reveal immune escape capability, and the high-risk group obtained higher scores than the low-risk one **Figure 8G**, in line with prior research results (54). The treatment of immune checkpoint blockers (ICBs) has made significant progress in HCC therapy, and predictors like CD8 and PD-L1 were used for the assessment of immune response. **Figures 8H, I** show the risk scores for CD8, PD-L1 and immune checkpoint molecules, and low- and high-risk groups were not significantly different.

### Establishment of a stemness-metabolism-related model based on risk scores

**Figure 9:**
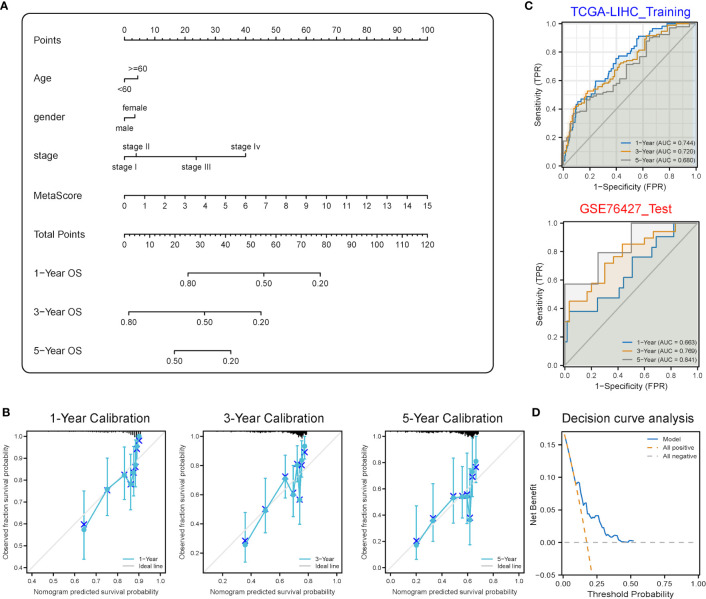
Predictive power analysis of metabolic risk scores related to mRNAsi on the HCC prognosis of patients. **(A)** Nomogram of the clinical prediction model on the basis of mRNAsi-related metabolic risk score combined with clinicopathological characteristics. **(B)** Calibration curve of the nomogram: Horizontal and vertical coordinates are the survival obtained from prediction and the actual observed survival, respectively. The nomogram showed good prognosis value for one-, three- and five-year survival. **(C)** Time-dependent ROC curve for training and validation sets, showing strong discrimination between training and validation set models. **(D)** DCA curve for the model, with y- and x-axis representing the net benefit and the probability of death, respectively. Brown and grey dashed lines represented the hypotheses that death occurs in all patients and no patients experience death, respectively.

A stemness-metabolism-related model combining the risk scores and clinicopathological features of HCC patients (such as gender, age and TNM staging) was established to predict survival rate (**Figure 9A**) and visualized by nomogram. Model accuracy was analyzed by calibration curves and the one-, three- and five-year survival probability forecast by the nomogram was closely bound up with the survival probability observed, confirming that the model was reliable (**Figure 9B**). After that, the ROC curve based on time was employed to calculate the AUC values of training (TCGA-LIHC) and validation datasets (GSE76427) (**Figure 9C**). All AUCs showed satisfactory results, demonstrating that the nomogram had excellent discrimination and could be applied to other cohorts. **Figure 9D** shows the decision curve analysis (DCA) curve of the model.

## Discussion

Heterogeneity is an essential and distinct feature of HCC and one of the main causes of poor prognosis. Staying in distinct differentiation states, CSCs maintain the ability to differentiate into diverse tumor cells, which contributes to heterogeneity. Accordingly, the subgroup classification based on CSCs might become a new viable way and shed light on future treatments. It is difficult to describe and quantify CSCs and stemness. Malta et al. adopted an innovative OCLR algorithm and defined mRNAsi as a new signature quantitatively reflecting the degree of oncogenic dedifferentiation (10). Since then, mRNAsi has provided new ideas and possibilities for linking stemness characteristics to clinical prognosis, gene mutations, treatment resistance, tumor immune characteristics, etc. Several studies have further investigated mRNAsi-related genes (55) and probed into the role of mRNAsi in HCC. Zhang et al. demonstrated a survival model using five mRNAsi-related genes (56), and Cai et al. constructed a six-gene prognosis signature (57). Mai et al. developed an HCC stemness risk model as a potential indicator of TACE treatment response (17). Zhang et al. and Xu et al. revealed the role that mRNAsi played in predicting immunotherapy response (58, 59). All studies obtained the same result that higher mRNAsi were correlated with worse outcomes and more advanced clinical stages. This is in accordance with the theory that CSCs are involved in the progression, recurrence, metastasis and treatment resistance of HCC. Because of technical limitations, not all mRNAsi-related genes are suitable and practical drug targets at present. Numerous cellular metabolic target drugs such as gemcitabine, 5-fluorouracil, l-asparaginase and methotrexate (60) have already addressed encouraging anti-cancer therapeutic effects in clinical practice or preclinical experiments. As a result, it was hypothesized that genes related to both stemness and metabolism reprogramming might be appropriate drug target candidates.

Metabolic signatures play a crucial role in tumor subclassification and immunotherapy response prediction. Some previous research had confirmed that metabolic signatures showed good power in tumor sub-phenotype and treatment response prediction in diffuse large B-cell lymphoma (61), ovarian (62) and colorectal cancers (63) as well as clear cell renal cell carcinoma (64). Chen et al. (65) made use of 90 metabolic genes to classify HCC and affirmed that metabolic signatures also showed great power in HCC subclassification. Three subtypes were developed: C1, C2 and C3 with high, low and intermediate metabolic activities, respectively. Further analysis was demonstrated, and the results showed that different subtypes also possessed different distinctions in prognostic value, immune infiltration and clinical characteristics. To be specific, C1 displayed low AFP expression and good clinical outcomes; C2 exhibited high-expression immune checkpoint inhibitors (ICIs), predicting high sensitivity to immunotherapy; C3 demonstrated high AFP expression and the worst prognosis (65). However, 90 genes for a classifier were too costly in clinical practice. In the present study, significant metabolic differences between low- and high-mRNAsi groups were confirmed. Besides, the association of metabolism reprogramming with clinical outcomes was revealed, with the enrichment of lipid-related metabolism pathways showing a relationship with poorer prognosis and carbon- and nitrogen-related metabolism pathways being closely related to better outcomes. The scope was narrowed, and a stemness-metabolic-gene signature with fewer genes was constructed. Additionally, clinical feasibility was improved while maintaining accuracy and sensitivity.

In this study, higher mRNAsi values were correlated with advanced clinical stages and worse clinical outcomes, which also aligned with previous research results (54, 59, 66). Subsequently, 74 DEMRGs were identified by performing differential analysis and WGCNA between low- and high-mRNAsi groups. LASSO and univariate cox regressions were explored, and the three most efficient prognostic genes, including PFKP, PDE2A and UGT1A5, were identified. PFKP is a protein-coding gene that translates into PFKP, an isoform of the rate-limiting enzyme of glycolysis, phosphofructokinase 1 (PFK1), which catalyzes the irreversible conversion of fructose-6-phosphate to fructose-1,6-biphosphate (67). As the important enzyme of glycolysis, PFKP was observed to allow cancer cells to survive under metabolic stress (68). Studies showed that PFKP went through an increase in HCC tissues (69) and was proven to be highly participated in glycolysis remodeling and associated with overall survival in HCC (70, 71). In addition to metabolic reprogramming, PFKP has a close correlation with stemness as well. PFPK served an essential role via LIF-Stat3 signaling to maintain embryonic stem cell (ESC) differentiation (72). The silencing of PFKP decreased the levels of stemness markers and proliferation capabilities in HCC (69). PFKP also played an important role in immune regulation. PFKP influenced stimulation of monocytes with oxidized low-density lipoprotein (73) and the expression level was correlated with interferon-gamma (IFN-γ) expression level (74). Sirtuin 2-PFKP interaction led to decreased light chain-3B activation and repressed phagocytosis (75). PFKP induced PD-L1 expression through EGFR activation and promoted immune evasion in human glioblastoma cells (76). In addition, a study explored the difference between progression HCC patients and partial response/stable HCC patients in response to the first-line combined immunotherapy, and PFKP showed a great difference in the level of mRNA, suggesting its potential in immunotherapy response stratification as well (77). PDE2A is a protein-coding gene that encodes PDE2A, an enzyme which belongs to the phosphodiesterase (PDE) family and hydrolyzes both 3’,5’-cyclic guanosine monophosphate (cGMP) and 3’,5’-cyclic adenine monophosphate (cAMP), mediating crosstalk between cGMP and cAMP signaling cascades (78), regulating mitochondrial clearance (79) and mitochondrial morphology (80, 81). The expression of PDE2A is tissue-specific and PDE2A is widely expressed in the brain and liver (78). PDE2A was demonstrated to correlate with tumorigenesis in osteosarcoma (82) and colorectal cancer (83) and to correlate with cancer stem cell stemness in glioma (84) and HCC (85). A recent study revealed that overexpressed PDE2A was associated with serum AFP level, vascular invasion, histologic grade, and pathologic stage, closely participating in inhibiting HCC cell proliferation, migration, and immune function, which had the potential to be used as a biomarker for HCC prognosis (81), the results of which consisted with our results. In glioma, PDE2A/miR-139 suppressed Wnt/β-catenin signaling by inhibiting cAMP accumulation and Glycogen Synthase Kinase-3β phosphorylation, thereby modulating the self-renewal of glioma stem cells (84). UGT1A5 is a protein-coding gene translated to UGT1A5, a member of the UGT1 family which is mainly implicated in the glucuronidation of bilirubin and phenol and acts as an essential player in the detoxification and metabolism (86, 87). Previous studies had reported UGT1A5 with a relatively low expression level in liver tissues and low enzymatic activity (88–91), while hepatic UGT1A5 expression was also proven highly inducible by multiple activators. UGT1A5 showed a significant age-dependent transcription in children (92). Treatment with rifampicin or 3-methylcholanthrene increased UGT1A5 expression level in human hepatocytes (86). In female efflux transported knockout FVB mice, the expression level of UGT1A5 was severely decreased, which indicated that UGT1A5 expression might be female-predominant at least for mice (93, 94). In the cholestasis mice model, the UGT1A5 expression level changed significantly as well (95). Those three genes were used as the basis for the establishment of a risk score model according to which patients with HCC were segmented into low- and high-risk groups. Differential analysis was performed. Moreover, it was further confirmed that DEGs were mainly involved in important metabolic pathways such as ether lipid, one-carbon and nitrogen metabolism, and disease associated with surfactant metabolism. Though the roles of PFKP, PDE2A and UGT1A5 in HCC weren’t fully understood yet, previous studies and our study suggested that the mechanisms of PFKP, PDE2A and UGT1A5 deserve further investigation. At last, the validation of the risk score model was completed in the GSE76427 dataset independently, and its AUCs corresponding to one-, three- and five-year survival were 0.740, 0.679 and 0.664, respectively, showing high predictive value.

Atezolizumab plus bevacizumab showed meaningful survival benefits in HCC, with a median OS of 19.4 months, further laying out the significance of immunotherapy in HCC treatments (96). Hence, gene signatures linked with the tumor microenvironment and the patterns of immune cell infiltration were analyzed as well, which could serve as important biomarkers to predict immunotherapy response. It has been reported that the tumor microenvironment could be conducive to maintaining CSCs which could modulate the tumor microenvironment and vice versa (21). In this study, the high-risk group demonstrated elevated immune and stromal scores, which meant that stemness features were positively associated with the abundance of stromal and immune cells. Compared with the low-risk group, the high-risk one exhibited an increased number of Tregs, M0 and other infiltrating suppressive immune cells, and higher expression levels of several immunotherapy target molecules, providing support for a negative association between stemness and immunotherapy efficacy. A recent study reported by Zhen Zhang et al. (58) demonstrated that stemness was robustly associated with ICIs through a different analysis method. This is in agreement with the findings of this research that cancer stemness was positively related to ICI resistance and intratumor heterogenicity.

Cumulative data from previous studies delineated an accurate picture of genetic variations in HCC and proved the correlation between gene alterations and antitumor immunity and metabolism (97). The genetic alterations of glucose metabolism, such as glucose-6-phosphatase, catalytic (G6PC), maturity-onset diabetes of the young type 3 (MODY3) and hepatocyte nuclear factor-1 alpha (HNF1A) genes, lead to glycogen storage diseases. That is, specific MODY3 could facilitate the occurrence of genetic liver adenomatosis and transform it into malignant HCC (98, 99). Low- and high-risk groups were found to have the same top gene mutations: tumor protein P53 (TP53), catenin beta-1 (CTNNB1) and titin (TTN) but different TMB and MSI levels and CNV patterns. The high-risk group showed more genomic alterations, indicating more possibility for immunotherapy resistance.

Finally, a nomogram making a combination of gender, age, TNM staging and gene signatures (PFKP, PDE2A and UGT1A5) was constructed for prognosis prediction and the predicted survival probability was closely fitted to the ideal line, indicating good efficacy.

CSCs are responsible for poor clinical outcomes yet are resistant to the majority of current therapies. Therapies capable of eliminating CSCs have aroused great concern, and many efforts have been dedicated to CSC-targeted therapies. CSC biomarkers are promising therapeutic targets, oncolytic measles viruses targeting CD133^+^ cells and EpCAM/CD3 and CD44 antibodies were invented in HCC (100–102). Nonetheless, no single biomarker is presented in all CSCs. For this reason, targeting a single biomarker resulted in the evasion of some CSCs (19, 103). Compared with biomarkers, CSCs share more biological features, with similar metabolic alterations. On this account, targeting altered metabolism-related pathways might be a better option and some drugs with metabolic targets have achieved inspiring results in preclinical and clinical studies. Metformin and Phenformin both restrain electron transport chain complex I and impair mitochondrial energy metabolism, giving rise to cell death in CSCs in pancreatic cancer (104). The down-regulation of the mammalian target of rapamycin (mTOR) signaling pathway which has been demonstrated to be deeply involved in energy homeostasis could reduce CSCs in breast cancer (105). The connection between metabolism reprogramming and stemness was uncovered in this study. Worthy of more in-depth research, DEMRGs have the potential to become targets for novel therapeutics. Moreover, a prognostic model containing both stemness and metabolic features was established before.

Nevertheless, limitations exist in this study. Statistical power was probably low on account of the relatively low sample size in both training and validation datasets. Therefore, the increase of statistical power makes it necessary to verify the predictive value of the novel stemness- and metabolism-related model by more HCC patients in the future. In clinical practice, BCLC staging is more widely used than TNM staging in HCC, but due to the lack of relevant clinical information on BCLC staging in public databases, our prediction model used TNM staging instead. More importantly, further experimental validations of PFKP, PDE2A, and UGT1A5 remain to be conducted at organismal, cellular and molecular levels despite powerful microarray-based bioinformatic analysis.

To sum up, the connection between metabolism reprogramming and cancer stem cells was comprehensively elucidated in this work. In addition, a survival model was established to predict prognosis and immunotherapy response with high accuracy, sensitivity and specificity. It was postulated that three genes could also be the potential therapeutic targets of HCC.

## Data availability statement

The datasets presented in this study can be found in online repositories. The names of the repository/repositories and accession number(s) can be found in the article/**Supplementary Material**.

## Author contributions

YW conceived the project and wrote the manuscript. XW participated in data analysis. SD reviewed the manuscript and participated in language editing. All authors contributed to the article and approved the submitted version.
